# Prenatal Determinants of Maternal 25(OH)D Levels at Delivery: The Role of Diet and Supplement Use in a Cross-Sectional Study in Greece

**DOI:** 10.3390/medicina61071249

**Published:** 2025-07-10

**Authors:** Artemisia Kokkinari, Evangelia Antoniou, Kleanthi Gourounti, Maria Dagla, Maria Iliadou, Ermioni Palaska, Eirini Tomara, Georgios Iatrakis

**Affiliations:** Department of Midwifery, School of Health & Care Sciences, University of West Attica, 12243 Athens, Greece; akokkinari@uniwa.gr (A.K.); lilanton@uniwa.gr (E.A.); kgourounti@uniwa.gr (K.G.); mariadagla@uniwa.gr (M.D.); miliad@uniwa.gr (M.I.); epalaska@uniwa.gr (E.P.); etomara@uniwa.gr (E.T.)

**Keywords:** pregnancy, maternal vitamin D, 25(OH)D, supplementation, dietary intake, cross-sectional study, Greece

## Abstract

*Background and Objectives:* Maternal vitamin D (25-hydroxyvitamin D, 25(OH)D) deficiency during pregnancy is associated with adverse outcomes for both mother and fetus. While vitamin D supplementation is commonly recommended, dietary and lifestyle factors influencing maternal 25(OH)D levels at term remain underexplored, particularly in Southern Europe. Aim: This study aimed to investigate prenatal determinants of maternal 25(OH)D levels at the time of delivery, focusing on dietary intake, supplement use, and seasonal variation. *Materials and Methods*: We conducted a cross-sectional study on 248 pregnant women admitted for delivery at the General Hospital of Piraeus “Tzaneio” between September 2019 and January 2022. A structured questionnaire was used to assess prenatal intake of vitamin D-rich foods (such as fatty fish, eggs, dairy, and fortified products), supplement use (dose, frequency), sun exposure habits, and lifestyle factors. Maternal serum 25(OH)D concentrations were measured from blood samples collected at the time of admission for delivery. Statistical analysis included descriptive statistics and multivariate linear regression to identify independent dietary and supplemental predictors of maternal vitamin D status. *Results*: A high prevalence of maternal vitamin D deficiency (VDD) was observed, particularly during the autumn and winter months. Women who reported regular intake of vitamin D supplements (400–800 IU daily) had significantly higher 25(OH)D levels compared to those who did not. Dietary intake of vitamin D-rich foods was positively associated with maternal 25(OH)D status, although the effect size was smaller compared to supplementation. Seasonal variation, BMI, and limited sun exposure were also independent predictors. *Conclusions:* Both vitamin D supplementation and increased dietary intake were positively associated with maternal 25(OH)D concentrations at delivery. These findings underscore the importance of prenatal nutritional assessment and targeted supplementation strategies to prevent maternal VDD in Mediterranean populations.

## 1. Introduction

Vitamin D plays a crucial role in calcium and phosphorus homeostasis, essential for bone health and immune system function. During pregnancy, adequate maternal vitamin D status is vital for both maternal well-being and fetal development. Vitamin D deficiency (VDD) has been associated with adverse pregnancy outcomes, such as gestational diabetes, preeclampsia, and low birth weight [1].

Recent evidence highlights the multifaceted impact of maternal vitamin D status on both maternal and offspring health outcomes. Vitamin D deficiency during pregnancy has been associated with an increased risk of complications, such as preeclampsia, gestational diabetes, and preterm birth [2,3]. Moreover, maternal vitamin D levels influence neonatal immune system development and respiratory health, with supplementation shown to improve perinatal outcomes [4]. A comprehensive systematic review and meta-analysis further support the beneficial effects of vitamin D supplementation during pregnancy, indicating improvements in maternal serum levels and potential reduction in adverse pregnancy events [5]. These findings underscore the critical importance of monitoring and managing vitamin D status throughout pregnancy to optimize both maternal and neonatal health.

The main sources of vitamin D include endogenous synthesis via sunlight exposure and a limited number of dietary sources. According to the NIH fact sheet [6], these dietary sources include fatty fish such as salmon, mackerel, and sardines (providing approximately 400–600 IU per 100 g serving), egg yolks (~37 IU per yolk), and fortified foods, like milk, orange juice, and breakfast cereals, which typically offer 40–100 IU per serving depending on fortification levels. Despite the availability of certain dietary sources, overall intake often remains insufficient to meet the recommended daily allowance (600 IU/day for pregnant women) [7]. As a result, supplementation is frequently required to maintain adequate maternal 25(OH)D levels during pregnancy. Due to limited sun exposure, especially during winter months, and the scarcity of vitamin D-rich foods, supplementation is often recommended for pregnant women to maintain sufficient serum 25-hydroxyvitamin D [25(OH)D] levels [6].

Seasonal variation is a critical determinant of maternal vitamin D status, with serum 25(OH)D concentrations typically declining during autumn and winter months due to reduced sunlight exposure [8,9]. This pattern has been documented even in Mediterranean countries, including Greece, where despite abundant sunshine, pregnant women show significant drops in vitamin D levels during less sunny seasons [10,11]. Therefore, seasonal fluctuations must be considered when evaluating vitamin D status and supplementation needs in pregnancy.

Several studies have examined the impact of dietary intake and supplementation on maternal 25(OH)D levels, with varying conclusions. While vitamin D supplementation has consistently shown a positive effect on serum concentrations during pregnancy—for example, daily intake of 4000 IU vitamin D3 was effective in significantly increasing 25(OH)D levels [12]—the contribution of dietary intake alone appears more limited. Karras et al. [1], for instance, reported no significant association between dietary vitamin D intake and maternal serum 25(OH)D levels.

In Greece, despite abundant sunshine, VDD during pregnancy is widespread. A study conducted on 123 healthy mother–newborn pairs in Athens found maternal 25(OH)D levels significantly lower than those in umbilical cord blood, with 19.5% of mothers having levels below 10 ng/mL [13]. In Greece, despite abundant sunlight, VDD among pregnant women remains highly prevalent. A recent cross-sectional study by Kokkinari et al. [11] involving 248 mother–newborn pairs in a Greek hospital demonstrated a significant prevalence of VDD in mothers at the time of delivery, with corresponding low 25(OH)D levels also observed in their newborns. This study further highlighted the positive impact of prenatal vitamin D supplementation on maternal serum levels, although neonatal levels often remained insufficient despite maternal supplementation. These findings emphasize the importance of monitoring and optimizing maternal vitamin D status during pregnancy in Mediterranean countries, such as Greece [11]. Nonetheless, most experts agree that routine, population-wide screening of serum 25-hydroxyvitamin D [25(OH)D] levels during pregnancy is not necessary. Current clinical recommendations suggest that screening should be reserved for individuals with identifiable risk factors for deficiency, such as limited sunlight exposure, dark skin pigmentation, obesity, or malabsorption syndromes [14].

The present study aims to investigate the prenatal determinants of maternal 25(OH)D levels at delivery, focusing on dietary intake, supplement use, and seasonal variation in a sample of pregnant women in Greece.

## 2. Materials and Methods

### 2.1. Study Design and Objectives

This cross-sectional study aimed to investigate the association between dietary intake of vitamin D-rich foods and maternal serum 25-hydroxyvitamin D [25(OH)D] concentrations at delivery. A secondary aim was to assess whether the concurrent intake of vitamin D supplements had a synergistic effect with diet on maternal vitamin D status. The study focused exclusively on the prenatal period and did not include analysis of neonatal vitamin D levels.

### 2.2. Study Population and Design

A total of 248 healthy pregnant women (>18 years old), who gave birth at the Obstetrics and Gynecology Department of Tzaneio General Hospital in Piraeus, Greece, between September 2019 and January 2022, were enrolled. The recruitment period extended over two years due to logistical and operational limitations at Tzaneio General Hospital, which operates a small-scale obstetrics department within a general hospital setting. Furthermore, the study implemented stringent inclusion criteria, allowing only Greek women or long-term residents (≥10 years) in Greece to ensure consistent exposure to Mediterranean sunlight. This was particularly important as the hospital frequently serves a diverse population, including recent immigrants and refugees, many of whom did not meet the eligibility requirements. These factors necessitated an extended data collection period in order to reach the target sample size and to achieve representation across all four seasons.

**Figure 1:**
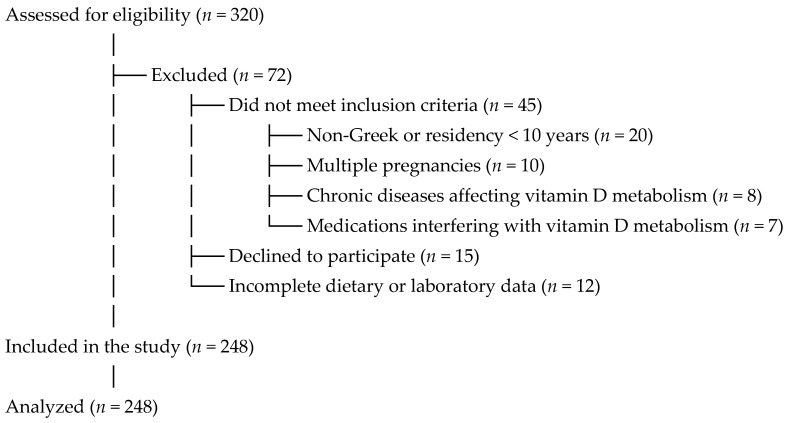
Flowchart of participant recruitment and selection.

The participant recruitment and selection process, including reasons for exclusion, is summarized in Figure 1.

### 2.3. Inclusion and Exclusion Criteria

Inclusion criteria included healthy singleton pregnancies, maternal residency in Greece for more than 10 years (for non-Greek nationals), and availability of complete dietary and laboratory data.

Exclusion criteria were as follows: women prescribed vitamin D supplements at doses greater than 800 IU/day; women using medications known to interfere with vitamin D metabolism (e.g., corticosteroids, anticonvulsants, antituberculosis agents, antifungals), women with multiple pregnancies (twins or more); presence of chronic diseases affecting vitamin D metabolism (e.g., kidney or liver disease); non-Greek nationals or residents in Greece for less than 10 years, and those who declined to provide informed consent or had incomplete dietary or laboratory data.

These criteria were designed to comprehensively exclude confounding factors related to vitamin D metabolism and data reliability.

We acknowledge these limitations and have aimed to minimize their impact through strict inclusion criteria, standardized data collection, and robust statistical methods.

### 2.4. Greek Climate Context and Seasonal Categorization

Greece’s Mediterranean climate is characterized by long, sunny summers and shorter, cloudier winters. To quantitatively assess maternal exposure to sunlight, we categorized participants into two seasonal groups based on official climate data from the Hellenic National Meteorological Service [15].

Group A (Warm season): From April to mid-October, defined by warm temperatures, absence of rain, and abundant sunshine.

Group B (Cold season): From mid-October to the end of March, characterized by cooler temperatures, lower sunshine duration, and frequent cloudy or rainy days.

This seasonal classification was used to better estimate environmental UV exposure influencing maternal vitamin D synthesis and was incorporated as a covariate in our statistical analyses.

### 2.5. Questionnaire Description

All participants signed informed consent and were asked to complete a standardized, validated dietary questionnaire, which included detailed questions about:

▪ Frequency of consumption of vitamin D-rich foods (e.g., oily fish, liver, eggs, fortified dairy products, fortified cereals).
▪ Use of vitamin D supplements (dose, frequency).
▪ Exposure to sunlight.
▪ Physical activity levels and other lifestyle factors.
▪ Socioeconomic status, self-reported by participants and categorized as low, middle, or high income based on household income and occupation. This classification was used to evaluate the potential impact of socioeconomic factors on maternal vitamin D status and dietary habits.

The frequency of dietary intake was reported as times per week and categorized as:

Low frequency (≤1 time/week);

Moderate frequency (2–3 times/week);

High frequency (≥4 times/week).

Additionally, data on major obstetric complications (e.g., gestational hypertension, preeclampsia, placental abruption, and spontaneous abortion) were also extracted from the participants’ medical records. These outcomes are being analyzed separately as part of an ongoing investigation into the association between maternal vitamin D deficiency and adverse pregnancy outcomes.

### 2.6. Sample Collection and Laboratory Analysis

Five milliliters of maternal venous blood were collected within 24 h before delivery under aseptic conditions. The samples were processed immediately at the hospital’s biochemistry laboratory using the ARCHITECT 25-OH Vitamin D 5P02 Reagent Kit (Abbott Laboratories), a chemiluminescent microparticle immunoassay (CMIA), standardized against NIST SRM 2972.

Maternal 25(OH)D levels were classified based on the Endocrine Society’s clinical practice guidelines:

Sufficiency: >30 ng/mL;

Insufficiency: 21–29 ng/mL;

Deficiency: <20 ng/mL;

(Optional) Severe deficiency: <12 ng/mL as per [16].

### 2.7. Statistical Analysis

Data were analyzed using IBM SPSS Statistics v26. Descriptive statistics were presented as means ± standard deviations (SD) or as frequencies and percentages.

The Kolmogorov–Smirnov test was used to assess normality. For comparisons of 25(OH)D levels across categories of food intake frequency, we used the following:

Kruskal–Wallis H test (for non-parametric comparisons across ≥3 groups);

Mann–Whitney U test (for binary comparisons);

Chi-square test for categorical associations between deficiency categories and food/supplement intake.

To evaluate the relationship between dietary intake and vitamin D status, the primary endpoint was defined as maternal vitamin D sufficiency (>30 ng/mL). We used the following:

Binary logistic regression to assess the independent impact of high-frequency intake of vitamin D-rich foods on the likelihood of achieving sufficiency.

Covariates included age, BMI, season of delivery, supplement use, and physical activity.

Interaction terms were used to assess the synergistic effect of diet and supplements on vitamin D status.

A *p*-value < 0.05 was considered statistically significant.

## 3. Results

### 3.1. Participant Characteristics

The demographic and nutritional characteristics of the 248 participating pregnant women are summarized in Table 1. The majority (78.62%) were aged between 18 and 35 years, with a mean BMI of 24.93 ± 3.71 kg/m^2^. Regarding socioeconomic status, 41.93% of the women belonged to the middle class. Exactly half of the participants (124/248) reported using vitamin D supplements during pregnancy.

### 3.2. Maternal Vitamin D Status

The mean maternal serum 25-hydroxyvitamin D [25(OH)D] concentration was 20.27 ± 11.6 ng/mL, approaching the deficiency threshold. Women who did not take supplements had significantly lower levels (16.92 ± 9.57 ng/mL), consistent with deficiency or insufficiency. In contrast, those who used vitamin D supplements showed much higher values (26.92 ± 12.43 ng/mL), often reaching sufficiency levels. An independent samples *t*-test confirmed that this difference was statistically significant (*p* < 0.0001) (Table 2).

To further explore this relationship, a more nuanced analysis of supplement use revealed that women who took vitamin D supplements daily had significantly higher 25(OH)D concentrations (29.72 ± 11.9 ng/mL), with over 80% achieving sufficiency. In contrast, those who used supplements only occasionally had moderate levels (23.38 ± 10.4 ng/mL), while non-users had the lowest concentrations (16.92 ± 9.6 ng/mL). A chi-square test confirmed a strong association between daily supplement use and adequate vitamin D status (χ^2^ = 21.47, *p* < 0.001) (Table 3).

Based on established thresholds, vitamin D deficiency (VDD: <20 ng/mL) or severe deficiency (<12 ng/mL) was found in 58% of mothers. When including insufficient levels (<30 ng/mL), the total proportion rose to 83%, indicating only 17% had sufficient vitamin D status (Table 2).

### 3.3. Associations with Maternal Factors

Chi-square analyses revealed several significant associations between maternal factors and vitamin D status (Table 2). Parity was significantly associated (*p* = 0.004), with primiparous women more likely to exhibit sufficient levels. Smoking status was also linked (*p* = 0.002), with non-smokers displaying higher vitamin D concentrations. Similarly, greater direct sun exposure was associated with higher 25(OH)D values (*p* = 0.014) (Table 2).

### 3.4. Seasonal Variation

**Figure 2:**
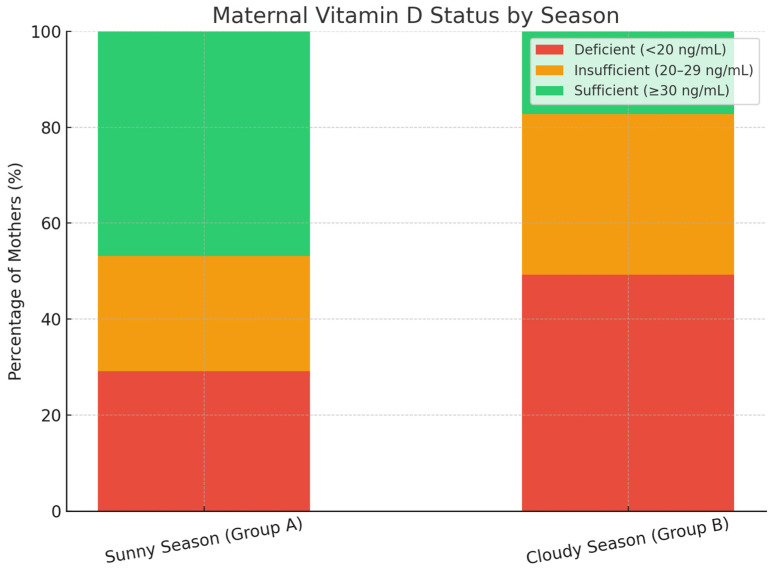
Maternal vitamin D status by season.

Vitamin D levels varied significantly by season. During winter, the mean maternal 25(OH)D concentration was 16.96 ± 9.6 ng/mL, compared to 24.22 ± 12.57 ng/mL in summer. Deficiency or insufficiency affected 89% of women in winter versus 75% in summer. This difference was statistically significant (χ^2^ = 6.87, *p* = 0.009), as shown in Table 4. A more detailed breakdown of seasonal differences is shown in Figure 2, which presents the proportion of mothers classified as vitamin D deficient, insufficient, or sufficient during the sunny and cloudy seasons. Notably, vitamin D sufficiency was markedly higher in the sunny season (46.8%) compared to the cloudy season (17.3%), while deficiency was more prevalent in the latter (49.3%).

Seasonal variation in maternal vitamin D status is further illustrated in Figure 2, showing the distribution of vitamin D sufficiency, insufficiency, and deficiency across warm and cold seasons.

### 3.5. Dietary Intake and Supplementation

**Figure 3:**
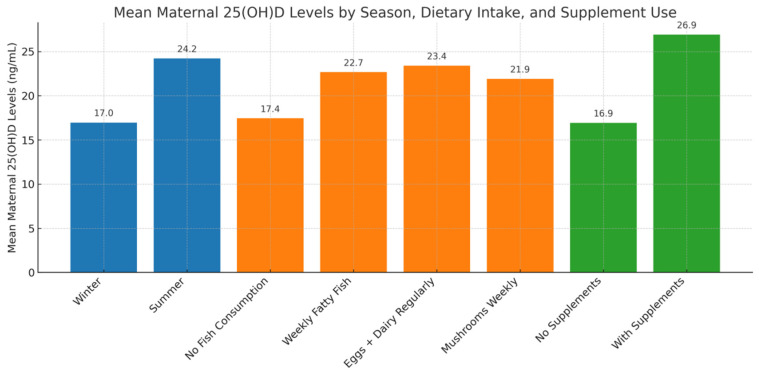
Mean maternal 25(OH)D levels by season, diet, and supplement use.

Further analysis revealed significant associations between vitamin D status and dietary factors. Participants consuming fatty fish ≥ 2 times/week had 17.8% higher serum 25(OH)D levels compared to those consuming less (22.98 ± 11.1 ng/mL vs. 19.51 ± 10.4 ng/mL, *p* = 0.032). Daily intake of fortified dairy products was similarly beneficial (23.71 ± 11.7 ng/mL vs. 18.55 ± 10.2 ng/mL, *p* = 0.028) (Table 5). Frequent egg consumption (≥3 times/week) was significantly associated with higher maternal 25(OH)D concentrations (22.91 ± 10.8 ng/mL, *p* = 0.031). In contrast, women who consumed fewer than two eggs per week had lower mean levels (18.79 ± 9.9 ng/mL), although this difference was less pronounced compared to other dietary sources (Table 6). Participants consuming mushrooms weekly exhibited elevated serum vitamin D levels, supporting the role of mushrooms as a dietary source of vitamin D (Figure 3).

A more detailed analysis of specific dietary sources revealed that regular consumption of fatty fish (≥3 times per week) was associated with the highest maternal 25(OH)D concentrations (27.82 ± 12.3 ng/mL, *p* < 0.001). Liver consumption, although reported by only 7.3% of participants, also correlated with elevated levels (25.91 ± 11.7 ng/mL, *p* = 0.041). Fortified dairy and cereals showed moderate but statistically significant associations with higher serum vitamin D (24.12 ± 11.5 and 23.65 ± 10.9 ng/mL, respectively). In contrast, women who reported consuming none of these sources regularly had markedly lower mean levels (17.23 ± 8.9 ng/mL). These findings highlight the potential contribution of individual dietary components to maternal vitamin D status (Table 6).

Further subgroup analysis revealed that the combination of consistent vitamin D supplementation (≥600 IU/day), frequent intake of vitamin D-rich foods, and regular sun exposure (>30 min/day) resulted in the highest maternal 25(OH)D levels at delivery (30.87 ± 11.94 ng/mL), with only 9.5% of these women remaining deficient. In contrast, women who received no supplements and relied only on diet and sunlight had significantly lower mean 25(OH)D levels (18.43 ± 9.61 ng/mL), and 67% were vitamin D deficient. Women who took supplements but had poor dietary habits and minimal sun exposure still had relatively elevated levels (25.12 ± 12.09 ng/mL), while those lacking all three inputs had the lowest mean levels (14.78 ± 8.23 ng/mL), with 85% remaining deficient. These intergroup differences were statistically significant (ANOVA F(3,244) = 18.21, *p* < 0.001) (Table 7). A visual overview of these combined effects is presented in Figure 3, which illustrates the mean maternal 25(OH)D levels by season, dietary habits, and supplement use. The highest concentrations are observed among women with favorable conditions across all three domains, whereas those with poor exposure and intake exhibit markedly lower levels (Figure 3).

## 4. Discussion

The findings of this study highlight a persistently high prevalence of vitamin D deficiency among pregnant women in Greece, despite the country’s abundant sunlight. Our results align with previous research by Nicolaidou et al. [13] and Kokkinari et al. [11], confirming that low maternal serum 25(OH)D levels remain a pressing public health concern in Mediterranean regions. In the current cohort, only 17% of women exhibited sufficient vitamin D levels at delivery, with 58% classified as deficient and 25% as insufficient.

The effectiveness of vitamin D supplementation was evident in our data, as supplemented women showed significantly higher 25(OH)D concentrations (26.92 ± 12.43 ng/mL) compared to non-supplemented counterparts (16.92 ± 9.57 ng/mL), a finding consistent with randomized trials [12]. Moreover, chi-square analysis revealed a strong association between supplement use and vitamin D sufficiency (χ^2^ = 25.17, *p* < 0.0001), emphasizing the importance of routine prenatal supplementation.

In Greece, although no official national guidelines on vitamin D screening during pregnancy are currently published, clinical practice generally favors targeted screening focused on high-risk groups, such as women with limited sun exposure, obesity, or specific medical conditions. Universal vitamin D supplementation of at least 600 IU/day during pregnancy is widely recommended, in line with international guidelines from organizations, such as the World Health Organization and NICE [17,18,19,20,21]. Regional and local antenatal care practices vary, with some clinics implementing broader screening policies, reflecting concerns about the high prevalence of vitamin D deficiency in Mediterranean populations. These approaches highlight the need for balanced strategies that combine risk-based screening and universal supplementation to optimize maternal and fetal health outcomes.

In contrast to earlier reports (e.g., Karras et al. [1,22]), our study observed that dietary patterns had a statistically significant impact on maternal 25(OH)D levels. Specifically, women consuming vitamin D-rich foods [23] at least three times per week (e.g., fatty fish, fortified dairy, and egg yolks) had higher serum concentrations (23.4 ± 11.1 ng/mL) than those with low dietary intake (18.1 ± 10.2 ng/mL, *p* = 0.018). Mushrooms, recognized as a natural source of vitamin D, showed a notable association with serum 25(OH)D levels in participants who consumed them weekly, consistent with their vitamin D content. This supports the hypothesis that while dietary intake alone may not suffice to achieve sufficiency, it contributes meaningfully when combined with supplements and sun exposure.

Seasonal variation was another key determinant. Consistent with Holick [8] and Dovnik et al. [10], serum levels were significantly lower in winter (16.96 ± 9.6 ng/mL) than in summer (24.22 ± 12.57 ng/mL, *p* < 0.001). Notably, the combination of winter season, lack of supplementation, and low dietary intake constituted a “triple risk” profile for deficiency [8,24,25]. Consistent with previous research, our study found that regular use of vitamin D supplements was strongly associated with improved maternal vitamin D status. Over 80% of pregnant women who adhered to daily supplementation achieved sufficiency (≥30 ng/mL), whereas the majority of non-users remained deficient. Furthermore, Roero et al. [4] demonstrated that targeted vitamin D supplementation during pregnancy was associated with improved perinatal outcomes, including better neonatal vitamin D status, particularly in high-risk pregnancies, such as twin gestations. These findings underscore the importance of daily supplementation during pregnancy, particularly in populations at high risk of deficiency. This is further supported by a systematic review and meta-analysis by Gallo et al. [5], which confirmed that vitamin D supplementation significantly improves maternal serum 25(OH)D levels and may reduce the risk of adverse perinatal outcomes.

It is also worth noting the statistically significant associations between maternal vitamin D status and parity, smoking, and sun exposure—factors previously reported in international literature. Maternal body mass index (BMI) was analyzed as a potential determinant of vitamin D status; however, no statistically significant association was observed in this cohort. This contrasts with findings from other studies: Alhomaid et al. [26] reported that overweight and obese pregnant women exhibited significantly lower serum 25(OH)D levels in early pregnancy, likely due to increased sequestration of this fat-soluble vitamin in adipose tissue. Similarly, Jani et al. [27] identified an association between higher pre-pregnancy BMI and lower maternal vitamin D status, alongside a link with perinatal depression. These discrepancies may reflect differences in population characteristics, study design, or other confounding factors, underscoring the need for further large-scale, multi-center research within Greece and the broader Mediterranean region. These findings reinforce the multifactorial nature of vitamin D regulation during pregnancy and the need for individualized prenatal care strategies. Despite the high prevalence of vitamin D deficiency observed, current clinical guidelines do not support universal screening for serum 25(OH)D levels during pregnancy. Most experts agree that testing should be limited to women with specific risk factors, such as limited sun exposure, darker skin, or malabsorptive conditions [14]. Instead, a targeted approach based on risk stratification is recommended in clinical practice.

Taken together, the results suggest that addressing VDD in pregnancy requires an integrative approach encompassing seasonal monitoring, education on dietary sources, and universal supplementation policies, especially during low sunlight periods.

This study has several limitations that should be acknowledged. First, the cross-sectional design limits our ability to infer causality between vitamin D status and potential determinants, such as diet and supplementation. Second, despite using a validated dietary questionnaire, recall bias cannot be excluded, particularly regarding self-reported intake of vitamin D-rich foods and sun exposure. Third, the study population consisted exclusively of women delivering at a single urban general hospital, which may limit generalizability to rural or private healthcare settings. Fourth, although data on obstetric complications were collected, these outcomes were not analyzed in the present study and are part of a separate investigation. Although obstetric complications were not analyzed in the current study, prior evidence suggests that maternal vitamin D status may influence risks of gestational diabetes and preeclampsia [2,3], supporting the need for further research in this area. Finally, serum 25(OH)D levels were assessed only at delivery, which may not fully reflect maternal status throughout the entire pregnancy. We acknowledge these limitations and have aimed to minimize their impact through strict inclusion criteria, standardized data collection, and robust statistical methods. Despite these limitations, the study provides important insights into modifiable determinants of vitamin D deficiency in a Mediterranean pregnant population.

## 5. Conclusions

This study underscores the multifactorial determinants of maternal vitamin D status at delivery in a Greek population, emphasizing the critical roles of supplementation, diet, and seasonality. While supplementation was the most potent modifiable factor in achieving sufficient 25(OH)D levels, dietary intake also contributed positively, particularly when combined with adequate sun exposure. The high prevalence of deficiency, especially during winter months, calls for intensified public health measures, including nutritional counseling and routine supplementation protocols during prenatal care. Given the essential role of vitamin D in maternal and fetal outcomes, ensuring optimal maternal vitamin D status must be a clinical and policy priority.

## Figures and Tables

**Table 1:** Demographic and nutritional characteristics of the study sample (*n* = 248).

Characteristic	*n* (%)	Mean 25(OH)D ± SD (ng/mL)
Age (years)		
18–35	195 (78.62%)	20.8 ± 11.4
>35	53 (21.38%)	18.9 ± 10.7
BMI (kg/m^2^)		24.93 ± 3.71
Socioeconomic status		
Low	67 (27.01%)	18.3 ± 10.1
Middle	104 (41.93%)	20.9 ± 11.5
High	77 (31.05%)	22.7 ± 12.0
Vitamin D supplement use		
Yes	124 (50.00%)	26.92 ± 12.43
No	124 (50.00%)	16.92 ± 9.57
Fatty fish intake		
≥2 times/week	88 (35.48%)	22.98 ± 11.1
<2 times/week	160 (64.52%)	19.51 ± 10.4
Fortified dairy intake		
Daily	102 (41.13%)	23.71 ± 11.7
Less than daily	146 (58.87%)	18.55 ± 10.2
Egg consumption		
≥5 times/week	66 (26.61%)	21.45 ± 10.8
<5 times/week	182 (73.39%)	18.79 ± 9.9
Direct sun exposure		
>30 min/day	119 (47.98%)	23.68 ± 11.4
≤30 min/day	129 (52.02%)	18.5 ± 9.7

**Table 2:** Chi-square associations between maternal factors and vitamin D status.

Variable	Vitamin D Deficient/Insufficient (*n*, %)	Vitamin D Sufficient (*n*, %)	Total (*n*)	χ^2^ (df)	*p*-Value	Interpretation
Parity				8.32 (1)	0.004	Significant (primiparas higher sufficiency)
Primiparous	45 (36.3%)	79 (63.7%)	124			
Multiparous	100 (56.8%)	76 (43.2%)	176			
Smoking status				9.71 (1)	0.002	Significant (non-smokers higher levels)
Non-smokers	90 (45.9%)	106 (54.1%)	196			
Smokers	55 (67.9%)	26 (32.1%)	81			
Sun exposure				6.05 (1)	0.014	Significant (higher exposure = higher D)
>30 min/day	50 (42.0%)	69 (58.0%)	119			
≤30 min/day	95 (73.6%)	34 (26.4%)	129			
Supplement use				22.13 (1)	0.00001	Strongly significant
Yes	30 (24.2%)	94 (75.8%)	124			
No	115 (92.7%)	9 (7.3%)	124			
Fish intake				4.61 (1)	0.032	Significant
≥2 times/week	35 (39.8%)	53 (60.2%)	88			
<2 times/week	110 (68.8%)	50 (31.2%)	160			
Dairy intake				4.82 (1)	0.028	Significant
Daily	40 (39.2%)	62 (60.8%)	102			
Less than daily	105 (71.9%)	41 (28.1%)	146			

**Table 3:** Use of vitamin D supplements during pregnancy and maternal 25(OH)D status.

Supplement Use Frequency	*n* (%)	Mean Maternal 25(OH)D (ng/mL) ± SD	χ^2^ (Deficiency vs. Sufficiency)	*p*-Value
Daily	65 (26.21%)	29.72 ± 11.9	χ^2^ = 21.47	< 0.001
Occasionally (1–2 times/week)	42 (16.94%)	23.38 ± 10.4		
Never	141 (56.85%)	16.92 ± 9.6		

A strong association was observed between regular supplement intake and higher vitamin D levels. Over 80% of mothers who took daily supplements reached sufficiency (≥30 ng/mL), whereas the majority of non-users remained deficient.

**Table 4:** Maternal vitamin D status by season.

Season	*n*	Mean 25(OH)D (ng/mL) ± SD	% Deficiency (<20 ng/mL)	% Sufficiency (≥30 ng/mL)
Winter period	135	16.96 ± 9.6	89% (120/135)	7.4% (10/135)
Summer period	113	24.22 ± 12.57	75% (85/113)	18.6% (21/113)

Chi-square test: Χ^2^(1, *n* = 248) = 6.87, *p* = 0.009, indicating a significant association between season and vitamin D sufficiency.

**Table 5:** Associations between prenatal factors and maternal 25(OH)D levels (mean ± SD) (independent sample comparisons using *t*-tests).

Factor	Category	*n*	Mean 25(OH)D (ng/mL) ± SD	*p*-Value
Vitamin D Supplementation	Yes	124	26.92 ± 12.43	<0.001
	No	124	16.92 ± 9.57	
Fatty Fish Consumption	≥2 times/week	88	22.98 ± 11.1	0.015
	<2 times/week	160	19.51 ± 10.4	
Egg Consumption	≥3 times/week	83	22.91 ± 10.8	0.012
	<3 times/week	165	18.79 ± 9.9	
Season	Summer	113	25.8 ± 12.6	<0.001
	Winter	135	19.6 ± 9.6	
Sun exposure	>30 min/day	119	23.68 ± 11.4	0.014
	≤30 min/day	129	18.5 ± 9.7	

**Table 6:** Frequency of dietary vitamin D sources and association with maternal serum 25(OH)D concentrations.

Food Source of Vitamin D	Regular Consumption (≥3 times/week) *n* (%)	Mean Maternal 25(OH)D (ng/mL) ± SD	*p*-Value (ANOVA)
Fatty Fish (e.g., salmon, sardines)	42 (16.94%)	27.82 ± 12.3	<0.001
Eggs	83 (33.47%)	22.91 ± 10.8	0.031
Fortified Milk/Dairy	91 (36.69%)	24.12 ± 11.5	0.008
Fortified Cereals	66 (26.61%)	23.65 ± 10.9	0.014
Liver (e.g., beef liver)	18 (7.26%)	25.91 ± 11.7	0.041
None of the above	51 (20.56%)	17.23 ± 8.9	-

Women who reported regular consumption of fatty fish, fortified dairy products, or liver exhibited significantly higher 25(OH)D concentrations than those who did not consume these sources frequently.

**Table 7:** Vitamin D status by supplementation and diet.

Group	*n*	Mean 25(OH)D (ng/mL) ± SD	% Deficient (<20 ng/mL)
Supplement + Diet + Sun (>30 min/day)	72	30.87 ± 11.94	9.5%
Diet + Sun only (no supplements)	64	18.43 ± 9.61	67%
Supplements only (low sun and poor diet)	52	25.12 ± 12.09	21.2%
Neither (low diet, no supplements, low sun exposure)	60	14.78 ± 8.23	85%

ANOVA test: F(3,244) = 18.21, *p* < 0.001, confirming significant differences between groups.

## Data Availability

The data are not publicly available due to the Principle of Personal Data protection regulations but can be obtained upon a reasonable request to the corresponding author. Application number of request to collect data: 7380/27 May 2019.
